# Evaluating the impact of artificial intelligence-assisted image analysis on the diagnostic accuracy of front-line clinicians in detecting fractures on plain X-rays (FRACT-AI): protocol for a prospective observational study

**DOI:** 10.1136/bmjopen-2024-086061

**Published:** 2024-09-05

**Authors:** Alex Novak, Max Hollowday, Abdala Trinidad Espinosa Morgado, Jason Oke, Susan Shelmerdine, Nick Woznitza, David Metcalfe, Matthew L Costa, Sarah Wilson, Jian Shen Kiam, James Vaz, Nattakarn Limphaibool, Jeanne Ventre, Daniel Jones, Lois Greenhalgh, Fergus Gleeson, Nick Welch, Alpesh Mistry, Natasa Devic, James Teh, Sarim Ather

**Affiliations:** 1 Emergency Medicine Research Oxford, Oxford University Hospitals NHS Foundation Trust, Oxford, UK; 2 Oxford University Hospitals NHS Foundation Trust, Oxford, UK; 3 Nuffield Department of Primary Care Health Sciences, University of Oxford, Oxford, UK; 4 Clinical Radiology, Great Ormond Street Hospital for Children, London, UK; 5 Radiology, UCL GOSH ICH, London, UK; 6 NIHR Great Ormond Street Hospital Biomedical Research Centre, London, UK; 7 Radiology, University College London Hospitals NHS Foundation Trust, London, UK; 8 Canterbury Christ Church University, Canterbury Christ Church University, Canterbury, UK; 9 Nuffield Department of Orthopaedics, Rheumatology and Musculoskeletal Sciences (NDORMS), Oxford Trauma & Emergency Care (OxTEC), University of Oxford, Oxford, UK; 10 Frimley Health NHS Foundation Trust, Frimley, UK; 11 Gleamer SAS, Paris, France; 12 Patient and Public Involvement Member, Oxford, UK; 13 Department of Oncology, University of Oxford, Oxford, UK; 14 Liverpool University Hospitals NHS Foundation Trust, Liverpool, UK; 15 North West MSK Imaging, Liverpool, UK; 16 Nuffield Orthopaedic Centre, Oxford University Hospitals NHS Foundation Trust, Oxford, UK

**Keywords:** Artificial Intelligence, Diagnostic Imaging, Fractures, Closed, Emergency Service, Hospital, RADIOLOGY & IMAGING

## Abstract

**Abstract:**

**Introduction:**

Missed fractures are the most frequent diagnostic error attributed to clinicians in UK emergency departments and a significant cause of patient morbidity. Recently, advances in computer vision have led to artificial intelligence (AI)-enhanced model developments, which can support clinicians in the detection of fractures. Previous research has shown these models to have promising effects on diagnostic performance, but their impact on the diagnostic accuracy of clinicians in the National Health Service (NHS) setting has not yet been fully evaluated.

**Methods and analysis:**

A dataset of 500 plain radiographs derived from Oxford University Hospitals (OUH) NHS Foundation Trust will be collated to include all bones except the skull, facial bones and cervical spine. The dataset will be split evenly between radiographs showing one or more fractures and those without. The reference *ground truth* for each image will be established through independent review by two senior musculoskeletal radiologists. A third senior radiologist will resolve disagreements between two primary radiologists. The dataset will be analysed by a commercially available AI tool, BoneView (Gleamer, Paris, France), and its accuracy for detecting fractures will be determined with reference to the ground truth diagnosis. We will undertake a multiple case multiple reader study in which clinicians interpret all images without AI support, then repeat the process with access to AI algorithm output following a 4-week washout. 18 clinicians will be recruited as readers from four hospitals in England, from six distinct clinical groups, each with three levels of seniority (early-stage, mid-stage and later-stage career). Changes in the accuracy, confidence and speed of reporting will be compared with and without AI support. Readers will use a secure web-based DICOM (Digital Imaging and Communications in Medicine) viewer (www.raiqc.com), allowing radiograph viewing and abnormality identification. Pooled analyses will be reported for overall reader performance as well as for subgroups including clinical role, level of seniority, pathological finding and difficulty of image.

**Ethics and dissemination:**

The study has been approved by the UK Healthcare Research Authority (IRAS 310995, approved on 13 December 2022). The use of anonymised retrospective radiographs has been authorised by OUH NHS Foundation Trust. The results will be presented at relevant conferences and published in a peer-reviewed journal.

**Trial registration numbers:**

This study is registered with ISRCTN (ISRCTN19562541) and ClinicalTrials.gov (NCT06130397). The paper reports the results of a substudy of STEDI2 (Simulation Training for Emergency Department Imaging Phase 2).

**STRENGTHS AND LIMITATIONS OF THIS STUDY**

- This study uses a detailed artificial intelligence-assisted fracture detection algorithm with a National Health Service-derived dataset.
- A broad set of health professionals will be recruited as participants, including under-represented groups such as nurse practitioners and physiotherapists.
- The enhanced dataset will allow evaluation of a broad range of pathologies, including rare but significant fractures.
- The dataset will have an abnormally high disease prevalence (50%) to include a broad range of pathologies.
- The small number of readers may reduce the statistical power for comparison between professional groups.

## Introduction

 Missed fractures are a source of serious harm for patients attending the emergency departments (EDs) and represent the most common diagnostic error in that clinical setting.^1^ Almost 2 million fractures occur annually in the UK with a lifetime prevalence of nearly 40%,^2^ while 5.1% of all ED attendances are for fractures or dislocations. National Health Service (NHS) Resolution has identified that misinterpretation of plain radiographs was the most common error leading to a successful claim for negligent ED care, leading to significant impacts on the lives of affected patients.^3^ Reported consequences include death, disability, deformity, need for further or prolonged treatments, chronic pain, emotional distress and loss of trust in the health service.^4^ Furthermore, the need for further attendances and prolonged or corrective treatment leads to significant excess healthcare costs.^5^

Most acute fractures are diagnosed by ED clinicians using plain radiographs as the first-line imaging investigation (National Clinical Guideline Centre, 2016), a task which requires time, skill and expertise. However, few of the clinicians fulfilling this role have any formal image interpretation training, and they vary significantly in experience.^6^ Furthermore, a workforce shortage of radiologists in the UK means that they are rarely able to undertake the primary evaluation of plain radiographs in ED.^7^ The high service pressures in UK EDs combined with a highly transient workforce results in a busy and distracting clinical environment that predispose to error and missing fractures on plain radiographs. An estimated 3.3% of fractures are missed on initial interpretation by ED staff.^8^ The error rate is higher on radiographs interpreted outside daytime working hours, which suggests that fatigue, workload and shift patterns may impact clinician performance.^9^

**Figure 1:**
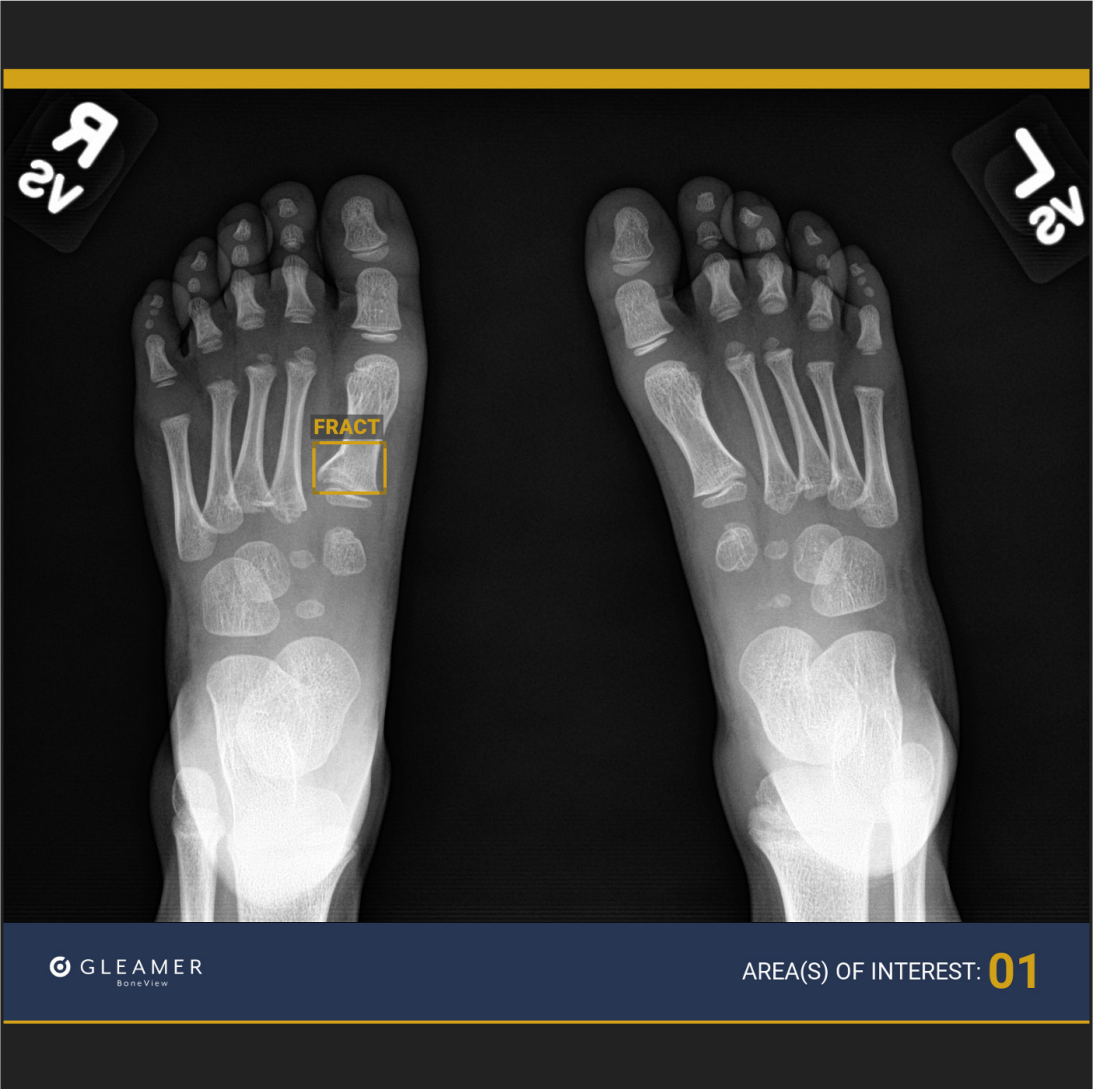
Image of Gleamer Boneview showing artificial intelligence-assisted overlay.

Over the last decade, advances in computer vision and machine learning have been used to augment interpretation of medical imaging.^10^ Several artificial intelligence (AI) algorithms have been developed that are able to detect fractures on plain radiographs with a high degree of accuracy.^11^ One such algorithm is the Gleamer BoneView (Gleamer, Paris, France) (see figure 1), which is currently the mostly widely used fracture detection algorithm in the NHS as well as worldwide (>800 sites in 30 countries). This algorithm estimates the likelihood of a fracture being present on a radiograph and provides users with three outcomes: *fracture*, no *fracture* and *uncertain*. If the likelihood has been estimated to be above a designated cut-off value, the area of abnormality is highlighted as a region of interest on a secondary image, which is made available to clinicians via their picture archive and communication system. If no abnormality is detected, this is also stated on the secondary image.^12 13^ Prior studies have demonstrated that the algorithm is highly accurate at detecting abnormalities, and it is already in use in a number of European centres, having received regulatory approval for use to support clinicians interpreting plain radiographs. Previous research has suggested that the algorithm is highly accurate at detecting abnormalities, and it is already in use in a number of European centres, having received regulatory approval for use to support clinicians interpreting X-rays. Moreover, recent studies have suggested that the use of AI software for detecting bone fractures^14 15^ can drastically decrease the rate of missed fractures. However, this software has not yet been fully tested in a UK setting using a locally derived dataset, and it is unclear to what degree such systems would affect the diagnostic performance of certain staff groups specific to the NHS, such as reporting radiographers and specialist nurse practitioners.

This study will evaluate the impacts of a commercially available AI-assisted image interpretation tool (Gleamer BoneView) on the diagnostic performance of the full range of clinicians (including nurses and allied health professionals) who routinely diagnose fractures in the NHS. It will address this evidence gap in the current evidence base, in line with the NICE (National Institute for Health and Care Excellence) Evidence Standards Framework for Digital Health Technologies, and recent Early Value Assessments which highlight the dearth of prospective evidence to support the use of AI-assisted image interpretation algorithms in the UK healthcare setting. Automation bias (the propensity for humans to favour suggestions from automated decision-making systems) is a known source of error in human-machine interaction^16^ and has been one of a number of causes for concern regarding the increasing usage of AI in radiology.^17^ A recent reader study in mammography,^18^ suggested significant automation bias presence across all levels of experience, noting that it was only the high-experienced reporters that consistently picked up on AI error. During our study, we will also assess the impact of incorrect advice given by the algorithm on the clinical end users.^19^

### Study aims

1. To evaluate the impact of AI-enhanced imaging on the diagnostic performance, efficiency and confidence of clinicians in detecting fractures on plain radiographs (primary).
2. To determine the stand-alone diagnostic accuracy of the BoneView AI tool with respect to the reference standard (secondary).
3. To determine associations between professional background and level of experience when determining the impact of AI support on clinician fracture detection (secondary).
4. To explore which imaging factors influence clinicians’ reporting accuracy and efficiency, and algorithm performance, for example, category of abnormality, size of abnormality, image quality, presence of multiple abnormalities (secondary).
5. To measure whether clinicians are more likely to make a mistake when AI provides an incorrect diagnosis (secondary).

## Methods and analysis

### Study design

**Figure 2:**
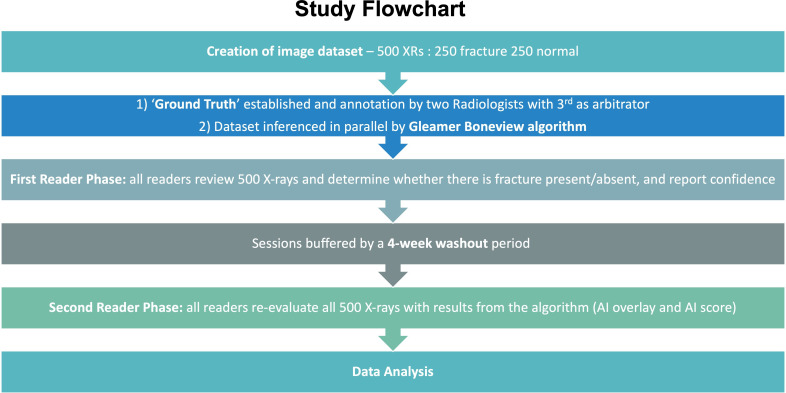
Study flowchart for artificial intelligence-assisted image analysis on the diagnostic accuracy of front-line clinicians in detecting fractures on plain X-rays multicase multireader study. AI, artificial intelligence; XRs, X-rays.

**Figure 3:**
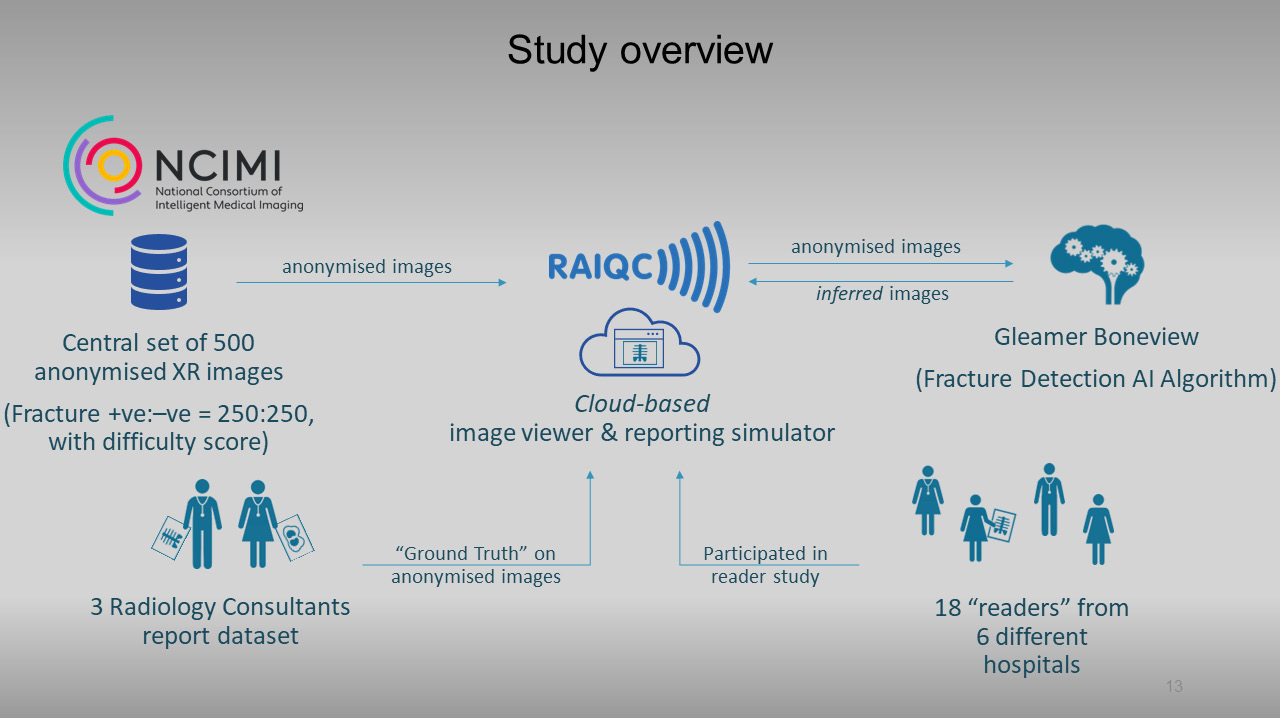
Artificial intelligence-assisted image analysis on the diagnostic accuracy of front-line clinicians in detecting fractures on plain X-rays study dataflows. AI, artificial intelligence; RAIQC, Report and Image Quality Control; XR, X-rays.

This study employs a multiple reader multiple case (MRMC) methodology. This approach involves multiple readers of various specialties and experience levels interpreting a large set of radiographs with and without AI assistance. The study processes are summarised in the flowchart in figure 2, with the dataflows represented in figure 3. The study design encompasses several key elements, including participant selection, case reading procedures, ground truthing process, case selection and AI algorithm inference on cases, which will be described in detail in the following subtitles.

### Participants

In order to explore the effects of using the algorithm on the full range of clinicians who diagnose fractures in routine practice and minimise selection bias, we have created a balanced matrix of readers in terms of specialty and seniority. 18 readers will be recruited from the following specialties (six specialities with three readers from each):

- Emergency physicians.
- Trauma and orthopaedic surgeons.
- Emergency nurses practitioners.
- Physiotherapists.
- General radiologists.
- Reporting radiographers.

Each specialty group will consist of a reader each fulfilling one of the following three levels of seniority:

- Consultant/senior/equivalent: >10 years experience.
- Registrar/equivalent: 5–10 years experience.
- Senior house officer/equivalent: <5 years experience.

Each specialty reader group will include one reader at each level of experience. Readers will be excluded if they have significant radiology experience in excess of their current specialty or grade. Prior use of fracture detection software does not exclude participation, as it is not expected in itself to confer a change in performance unless actively used during interpretation.

Readers will be recruited from across four NHS organisations that comprise the Thames Valley Emergency Medicine Research Network (www.TaVERNresearch.org):

- Oxford University Hospitals (OUH) NHS Foundation Trust.
- Royal Berkshire NHS Foundation Trust.
- Frimley Health NHS Foundation Trust.
- Milton Keynes University Hospital NHS Foundation Trust.

Participants will be recruited through a structured invitation process coordinated by the research team. A designated team member will collaborate with clinical leads and research coordinators at each participating site within the Thames Valley Emergency Medicine Research Network to identify potential participants based on predetermined criteria. These criteria include fulfilment of the required specialty and experience level categories, demonstrated commitment to professional development and research, and ability to commit to the full duration of the study.

All invitations will be extended based on the aforementioned criteria, and participation will be voluntary, maintaining objectivity throughout the recruitment process.

### Setting

The reads will be performed using a secure web-based DICOM viewer (www.raiqc.com). The platform allows readers to view radiographs and identify the site of an abnormality with a mouse click. The images will be viewable through a web browser on desktop or laptop devices, reflecting standard real-world hospital practice in which radiographs are typically interpreted by clinicians without dedicated high-resolution viewing stations.

Prior to beginning each phase of the study, the readers will undergo a training module that includes reading 5 practice images (not part of the 500-image dataset) to familiarise themselves with the use of the study platform and the output of the AI tool.

### Case selection and composition

The image dataset will include anonymised radiographs of adult patients (≥18 years) who presented to the EDs of OUH NHS Foundation Trust with a suspicion of fracture after injury to the limbs, pelvis or thoracolumbar spine. As CT is the investigation of choice for skull and many cervical spine injuries, these will be excluded from the study. Paediatric patients will be excluded from the dataset as their fracture types differ from those in adults, and there is an ongoing study evaluating this aspect (FRACTURE study; Fast Reporting using Artificial Intelligence for Children's TraUmaticRadiology Examinations^12^). Obvious fractures (defined as fractures including any of the following: displacement>5 mm, shortening>5 mm or angulation>5°) will also be excluded.

To constitute the dataset, radiology reports will be screened from the radiology information system to develop an enriched dataset of the 500 standard clinical examinations evenly split between normal and abnormal, with one or more fractures. The ratio of radiographs from each anatomical location has been informed by the proportion of missed fractures mentioned in the NHS Resolution report (table 1).

**Table 1:** Proportion of radiographs of each anatomical location, based on the proportion of missed fractures mentioned in the National Health Service Resolution report

Body part	Images in the dataset, n
Spine	42
Shoulder	20
Elbow	20
Wrist/hand	150
Hip/pelvis	130
Knee	42
Foot/ankle	96

To ensure a like-for-like comparison, image finding for abnormal cases will be performed first. The normal images will be age and sex matched per body part. We will aim to include representation of the different image views, system type (mobile or fixed), system vendors and patient demographics (eg, age, sex) without any prespecified quota.

The dataset will then be anonymised and uploaded to the Report and Image Quality Control platform under an existing data governance approval from the OUH NHS Foundation Trust Caldicott guardian.

### Case inclusion and exclusion summary

#### Inclusion

Plain radiographs of adult patients (age>18 years) presenting to the OUH ED with a suspected fracture.

#### Exclusion

- Plain skull radiographs.
- Plain cervical spine radiographs.
- Follow-up radiographs for known fracture.
- Paediatric radiographs (age<18).
- Obvious fractures defined as:
  - Displacement>5 mm.
  - Shortening>5 mm.
  - Angulation>5°.

### Inferencing the image dataset

The entire dataset of images will then be separately analysed using BoneView, creating a duplicate dataset of radiographs with alerts and regions of interest indicated.

### Radiographic interpretation

All readers will review all 500 radiographs individually across 2 reporting rounds.

In the first round, they will interpret the images as per clinical practice without any AI assistance. After a washout period of a month to mitigate the effects of recall bias, they will review the same 500 radiographs a second time with the assistance of the algorithm, which will contribute its suggestions as to abnormality presence and location. In both sessions, clinicians will be blinded to the ground truth established by the MSK (musculoskeletal) radiologists.

Clinician readers will be asked to identify the presence or absence of fracture by placing a marker on the image at the location of the fracture (if present) and to rank their confidence for fracture identification. Confidence rating will take the form of a Likert scale from 1 to 5 with 1 being least confident and 5 most confident.

### Ground truthing

The gold standard reference process will be conducted by two experienced musculoskeletal radiologists (>10 years’ experience) who will independently review and annotate each of the 500 radiographs in the dataset. They will draw bounding boxes around each detected fracture and grade the images on both image quality and difficulty of abnormality detection using a 5-point Likert scale.

In cases of disagreement between the two primary radiologists regarding the presence or absence of abnormalities, a third senior musculoskeletal radiologist will review the contentious images and make a final decision.

All annotations, gradings and arbitration decisions will be documented within the secure web-based DICOM viewer platform, establishing a reliable reference standard for evaluating both human reader performance and AI assistance.

In the event of significant discrepancies persisting after the initial arbitration process, a consensus meeting will be agreed. This meeting will include the primary ground truth radiologists, the arbitrator and key members of the research team. The purpose of this meeting will be to review and resolve any remaining discrepancies, ensuring the integrity and consistency of the final reference standard. This collaborative approach will be employed only for cases where substantial disagreement remains, thereby maintaining the overall objectivity of the ground truth process while addressing complex or ambiguous cases.

### Study timeline

This study commenced on 8 February 2024 and is actively collecting data. The data collection and analysis phase is projected to finish by the end of September 2024 with write up and publication anticipated later in the year.

### Outcome measures

Reader and AI performance will be evaluated using sensitivity, specificity, positive predictive value (PPV), negative predictive value (NPV) and area under receiver operating characteristic curve (AUC). Reader performance will be evaluated with and without AI assistance.

Reader speed will be evaluated as the mean review time per scan, with and without AI assistance.

Reader confidence will be evaluated as self-reported diagnostic confidence on a 5-point Likert scale, with and without AI assistance.

### Data statement and management

Radiographs selected for the study will be anonymised in accordance with OUH NHS Foundation Trust information governance protocol and uploaded to the secure image viewing platform (www.raiqc.com). Access to the radiographs will be controlled via the study platform using separate user accounts for each reader.

All study data will be entered into a password-protected and secure database. Individual reader accuracy scores will be anonymised, and the study team will not have access to the identifying link between the participants’ personal details and the data. Data about the participants’ seniority level and professional group will be retained to allow group comparisons.

### Sample size and power calculation

The study’s sample size of 500 images, evenly split between normal and abnormal cases, was determined using the Multi-Reader Sample Size Program for Diagnostic Studies. This tool, developed by Hillis,^20^ is specifically designed for MRMC study power calculations. Based on parameters derived from our previous MRMC study on pneumothorax detection, the programme calculated that with 18 readers and 500 cases, our study will achieve 85% power to detect a 10% difference in accuracy between unassisted and AI-assisted readings, with a 5% type 1 error rate (See output from software below).

The chosen sample size of 500 images ensures sufficient statistical power and adequate representation of fracture types and anatomical locations. This robust sample size, combined with our substantial and diverse reader pool, should enable the detection of clinically significant improvements in fracture detection accuracy and allow for subgroup analyses across specialties and experience levels. By using this rigorously calculated sample size, we aim to produce statistically robust and clinically relevant results that can inform the potential integration of AI assistance in fracture detection across various clinical settings, while adequately addressing our study objectives and maintaining statistical validity.

### Statistical analyses

The performance of the algorithm will be compared with the ground truth generated by the musculoskeletal radiologist panel. The continuous probability score from the algorithm will be used for the AUC analyses, while binary classification results with three different operating cut-offs will be used for evaluation of sensitivity, specificity, PPV and NPV. Sensitivity and specificity of readers with and without AI will be tested based on the Obuchowski-Rockette model for MRMC analysis which will model the data using a two-way mixed effects analysis of variance (ANOVA) model treating readers and cases (images) as random effects and effect of AI as a fixed effect with recommended adjustment to df by Hillis.^21^

The difference in diagnostic characteristics (sensitivity, specificity, accuracy, area under the receiver operating characteristic (ROC) curve) of readers as compared with ground truth with and without AI assistance will be the primary outcome on a per image and per abnormality basis. The main analysis will be performed as a single pooled analysis including all groups and sites. Secondary outcomes will include comparison between the performance of subgroups by specialty (emergency medicine, trauma/orthopaedics, physiotherapy, nurse practitioner, radiologist, radiographer), level of seniority (senior, middle grade, junior), degree of difficulty of the image and by anatomical region. Reader-reported confidence with and without the AI assistance will be compared. Secondary outcomes include the diagnostic characteristics of the AI algorithm alone. Surveys will be conducted throughout the study to measure the satisfaction, adoption and confidence in the AI algorithm of the study participants. Per-patient sensitivity will be defined as the proportion of reads in which all true fractures were marked as a proportion of the reads having at least one fracture. Per-patient specificity will be defined as the proportion of reads in which no fracture was marked by the reader as a proportion of the reads that did not show a fracture. These definitions disregard the detection of multiple fractures thus we will define the fracture-wise sensitivity as the proportion of fractures correctly detected as a proportion of all fractures. The two coprimary outcomes will be patient-wise sensitivity and patient-wise specificity. The stand-alone algorithm performance will be assessed by calculating the area under the curve (AUC) of the ROC and free-response ROC curves plotted with their variance. To account for correlated errors arising from readers interpreting the same images with and without AI, the Obuchowski and Rockette, Dorfman-Berbaum-Metz^22^ procedure; a modality-by-reader random effects ANOVA model will be used for estimation. Analyses will be carried out using R and the MRMCaov library.

### Strengths and limitations

This study uses a CE (Conformité Européenne)-marked AI-assisted fracture detection algorithm with an NHS-derived dataset. The enhanced dataset will allow evaluation of a broad range of pathologies, including rare but significant fractures and its composition is mapped to mirror the proportions of missed fracture locations seen in the NHS Resolution report. A broad set of health professionals will be recruited as participants, including under-represented groups such as nurse practitioners and physiotherapists, from multiple hospital sites across the region—these reflect a reader group not yet explored in the literature, and one directly applicable to the NHS.

In terms of limitations, while the overall study group is large in comparison to other similar reader studies, the small number of readers in subgroups may reduce the statistical power for comparison between professional groups. The dataset will include an abnormally high disease prevalence (50%) to include a broad range of pathologies to facilitate meaningful statistical comparison, meaning that while the reader study will effectively explore the impact of the algorithm on readers interpreting a broad and detailed dataset, the results will not mirror the prevalence of pathologies encountered in normal clinical practice and further prospective study will be required to determine efficacy in this regard.

### Patient and public involvement (PPI)

This protocol has been reviewed by the Oxford ACUTECare PPI group and PPI representatives on the artificial intelligence-assisted image analysis on the diagnostic accuracy of front-line clinicians in detecting fractures on plain X-rays steering group. They have supported the study and its aims, were involved in the grant application, design and data management stages and have advised on dissemination strategies.

### Ethics and dissemination

The study has been approved by the UK Health Research Authority (IRAS number 310995, approved on 13 December 2022). The use of anonymised retrospective radiographs has been authorised by the Caldicott Guardian and information governance team at OUH NHS Foundation Trust. Readers will provide written informed consent and will be able to withdraw at any time.

The study is registered at Clinicaltrials.gov (NCT06130397) and the ISRCTN (ISRCTN19562541) registry (approval pending reference 44612). The results of the study will be presented at relevant conferences and published in peer-reviewed journals. The detailed study protocol will be freely available on request to the corresponding author. Further dissemination strategy will be strongly guided by our PPIE (Patient and Public Involvement and Engagement) activities. This will be based on co-productions between patient partners and academics and will involve media pieces (mainstream and social media) as well as communication through charity partners. Key target audiences will include non-specialist clinicians routinely involved in fracture detection, as well as hospital managers, health policy-makers and academics working in AI-assisted image analysis.
