# Supplementary material for: Evaluating the impact of artificial intelligence-assisted image analysis on the diagnostic accuracy of front-line clinicians in detecting fractures on plain X-rays (FRACT-AI): protocol for a prospective observational study
Source: BMJ Open. 2024 Sep 5;14(9):e086061. doi: 10.1136/bmjopen-2024-086061 (PMC11381697; doi:10.1136/bmjopen-2024-086061)
Supplement: Supplementary file 1 [file bmjopen-2024-086061.reviewer_comments.pdf]

PEER REVIEW HISTORY

BMJ Open publishes all reviews undertaken for accepted manuscripts. Reviewers are asked to complete a checklist review form and are provided with free text boxes to elaborate on their assessment. These free text comments are reproduced below.

ARTICLE DETAILS

**TITLE (PROVISIONAL)**	Evaluating the impact of artificial intelligence-assisted image analysis on the diagnostic accuracy of frontline clinicians in detecting fractures on plain x-rays (FRACT-AI): protocol for a prospective observational study
**AUTHORS**	Novak, Alex; Hollowday, Max; Espinosa Morgado, Abdala Trinidad; Oke, Jason; Shelmerdine, Susan; Woznitza, Nick; Metcalfe, David; Costa, Matthew; Wilson, Sarah; Kiam, Jian Shen; Vaz, James; Limphaibool, Nattakarn; Ventre, Jeanne; Jones, Daniel; Greenhalgh, Lois; Gleeson, Fergus; Welch, Nick; Mistry, Alpesh; Devic, Natasa; Teh, James; Ather, Sarim

VERSION 1 - REVIEW

**REVIEWER NAME**	Mahamuni, Chaitanya Vijaykumar
**REVIEWER AFFILIATION**	Agnel Charities' Father Conceicao Rodrigues Institute of Technology
**REVIEWER CONFLICT OF INTEREST**	None
**DATE REVIEW RETURNED**	25-Mar-2024

**GENERAL COMMENTS**	1. Clarify the Study Design: Provide more detail on the specific design elements of the Multiple-Reader Multiple-Case (MRMC) study, including how cases will be selected, reader recruitment criteria, and the training module for participants.<br>2. Expand on Ground Truthing Procedures: Describe in greater depth the process for establishing ground truth for each image, including how discrepancies between radiologists will be resolved and how image difficulty will be assessed.<br>3. Increase Sample Size Justification: Provide a more robust justification for the chosen sample size of 500 images, particularly regarding statistical power calculations and how this sample size will adequately address the study objectives.<br>4. Enhance Data Analysis Section: Provide more clarity on the statistical methods and analyses planned for evaluating reader and AI performance, including how sensitivity, specificity, PPV, NPV, and AUC will be calculated and compared between different groups.<br>5. Include Automation Bias Discussion: Expand on the potential impact of automation bias in the interpretation of AI-assisted radiographs, including how this bias will be mitigated or accounted for in the study design and analysis.<br>6. Address Potential Biases: Discuss potential biases that may influence the study results, such as selection bias in reader recruitment or the impact of prior experience with AI algorithms on reader performance.<br>7. Improve Ethics and Dissemination Section: Provide more detail on the ethical considerations involved in the study, including any potential risks to participants and how they will be mitigated. Additionally, elaborate on the planned dissemination strategy, including specific target audiences and channels.

	8. Clarify Data Management Protocols: Provide a detailed explanation of the data management protocols, including how data will be anonymized, stored, and accessed by researchers. Ensure compliance with data protection regulations and guidelines.<br>9. Strengthen Limitations Section: Expand on the limitations of the study, including potential sources of bias, generalizability of findings to other settings, and the impact of any constraints or limitations on the study design.<br>10. Improve Clarity and Readability: Enhance the overall clarity and readability of the manuscript by streamlining complex sentences, using clear and concise language, and organizing information in a logical manner to improve comprehension for readers.

**REVIEWER NAME**	Sedaghat, Sam
**REVIEWER AFFILIATION**	University Hospital Heidelberg
**REVIEWER CONFLICT OF INTEREST**	None
**DATE REVIEW RETURNED**	29-Mar-2024

**GENERAL COMMENTS**	Interesting protocol study on evaluating the impact of artificial intelligence-assisted image analysis on the diagnostic accuracy of frontline clinicians in the detection of fractures on plain X-Ray. I have some comments:<br><br>Please check the order of your references. The first reference is No. 7<br><br>Please add a short future perspective and please discuss in a few words that future studies could deploy this strategy on more advanced imaging techniques for fracture detection like CT. Please consider adding these recently published article to your references: https://doi.org/10.1016/j.ejrad.2024.111364 and https://doi.org/10.3390/diagnostics13172745. The one is on AI in spine fractures, the other is on detecting fractures using novel imaging techniques. Interesting to point towards future studies combining your concept with further or innovative techniques.<br><br>Would also be good to add some images.

VERSION 1 – AUTHOR RESPONSE

Reviewer: 1
Dr. Chaitanya Vijaykumar Mahamuni, Agnel Charities' Father Conceicao Rodrigues Institute of Technology

Comments to the Author:

*Clarify the Study Design: Provide more detail on the specific design elements of the Multiple-Reader Multiple-Case (MRMC) study, including how cases will be selected, reader recruitment criteria, and the training module for participants.

Thank you for this comment – we have updated these sections as requested

*Expand on Ground Truthing Procedures: Describe in greater depth the process for establishing ground truth for each image, including how discrepancies between radiologists will be resolved and how image difficulty will be assessed.

Thank you for this comment – we have updated sections requested

*Increase Sample Size Justification: Provide a more robust justification for the chosen sample size of 500 images, particularly regarding statistical power calculations and how this sample size will adequately address the study objectives.

Thank you for this comment – we have updated the sample size calculation as suggested and aligned it to study objectives

*Enhance Data Analysis Section: Provide more clarity on the statistical methods and analyses planned for evaluating reader and AI performance, including how sensitivity, specificity, PPV, NPV, and AUC will be calculated and compared between different groups.

Thank you for this comment – we have updated this section accordingly

*Include Automation Bias Discussion: Expand on the potential impact of automation bias in the interpretation of AI-assisted radiographs, including how this bias will be mitigated or accounted for in the study design and analysis.

Thank you for this comment. This study is specifically measuring the performance of clinicians with and without AI, so measurement of automation bias is an integral part of the study. We will explore this further in a separate sub analysis which will identify which incorrect reads by participants were based on false positive and negative results, and how this varied by reader subgroup.

*Address Potential Biases: Discuss potential biases that may influence the study results, such as selection bias in reader recruitment or the impact of prior experience with AI algorithms on reader performance.

Thank you for this comment. We have added commentary on this to the design and methodology section of the paper.

*Improve Ethics and Dissemination Section: Provide more detail on the ethical considerations involved in the study, including any potential risks to participants and how they will be mitigated.

Additionally, elaborate on the planned dissemination strategy, including specific target audiences and channels.

Thank you for this comment - we have updated this section accordingly.

*Clarify Data Management Protocols: Provide a detailed explanation of the data management protocols, including how data will be anonymized, stored, and accessed by researchers. Ensure compliance with data protection regulations and guidelines.

Thank you for this comment. This is covered in our ‘Data statement and management section’.

*Strengthen Limitations Section: Expand on the limitations of the study, including potential sources of bias, generalizability of findings to other settings, and the impact of any constraints or limitations on the study design.

Thank you for this comment. At your suggestion we have added a section on strengths and limitations

*Improve Clarity and Readability: Enhance the overall clarity and readability of the manuscript by streamlining complex sentences, using clear and concise language, and organizing information in a logical manner to improve comprehension for readers.

Thank you for this comment – we have reviewed and edited the manuscript accordingly.

Reviewer: 2
Dr. Sam Sedaghat, University Hospital Heidelberg

Comments to the Author:

Interesting protocol study on evaluating the impact of artificial intelligence-assisted image analysis on the diagnostic accuracy of frontline clinicians in the detection of fractures on plain X-Ray. I have some comments:

*Please check the order of your references. The first reference is No. 7

Thank you for this comment – done as requested

*Please add a short future perspective and please discuss in a few words that future studies could deploy this strategy on more advanced imaging techniques for fracture detection like CT. Please consider adding these recently published article to your references: https://doi.org/10.1016/j.ejrad.2024.111364 and https://doi.org/10.3390/diagnostics13172745. The one is on AI in spine fractures, the other is on detecting fractures using novel imaging

techniques. Interesting to point towards future studies combining your concept with further or innovative techniques.

Thank you for this comment – done as requested

*Would also be good to add some images.

Thank you for this comment. We have added some images as suggested including sample overlays, study processes and dataflows to better explain the study.

Based on last email (July 18, 2024):

*Please provide a point-by-point response to the Editor's comments and reviewer's comments and submit them in Word Format.

Thank you for this comment – done as requested

*Please provide a more detailed contributorship statement. It needs to mention all the names/initials of authors along with their specific contribution/participation for the article. This should be stating how each author contributed to the article. It should discuss the planning, conduct and reporting of the work in your paper. You may also consider the conception and design, acquisition of data or analysis and interpretation of data, etc. The statement in the ScholarOne system and main document should be matched. Please see link below for more information: https://authors.bmj.com/policies/bmj-policy-on-authorship/

Thank you for this comment – done as requested

*Please also identify the guarantor in your Contributorship Statement in the main document and in ScholarOne system.
For example: The guarantor of the study is (Author name / Author initials); accepts full responsibility for the finished work and/or the conduct of the study, had access to the data, and controlled the decision to publish.'

Thank you for this comment – done as requested

We believe these revisions have significantly improved the quality and clarity of our manuscript. We look forward to your feedback and hope you find the revised version suitable for publication in BMJ Open.

Sincerely,

Prof Alex Novak

BSc (Hons) MBChB MSc MRCGP DRCOG FRCEM

RCEM Associate Professor of Emergency Medicine

Director of Emergency Medicine Research Oxford (EMROx)

Consultant in Emergency Medicine and Ambulatory Care

Oxford University Hospitals NHS Foundation Trust

Clinical Specialist Advisor, Trinity College Oxford

VERSION 2 – REVIEW

**REVIEWER NAME**	Mahamuni, Chaitanya Vijaykumar
**REVIEWER AFFILIATION**	Agnel Charities' Father Conceicao Rodrigues Institute of Technology
**REVIEWER CONFLICT OF INTEREST**	None
**DATE REVIEW RETURNED**	28-Jul-2024

**GENERAL COMMENTS**	The said corrections are made and the paper can be accepted for publication

**REVIEWER NAME**	Sedaghat, Sam
**REVIEWER AFFILIATION**	University Hospital Heidelberg
**REVIEWER CONFLICT OF INTEREST**	None
**DATE REVIEW RETURNED**	10-Aug-2024

**GENERAL COMMENTS**	Thank you for your changes that improved the manuscript significantly. However, the requested references have not been added to your manuscript. Also, I cannot find the requested perspective section:<br>"Please add a short future perspective and please discuss in a few words that future studies could deploy this strategy on more advanced imaging techniques for fracture detection like CT. Please consider adding these recently published article to your references: https://doi.org/10.1016/j.ejrad.2024.111364 and https://doi.org/10.3390/diagnostics13172745. The one is on AI in spine fractures, the other is on detecting fractures using novel imaging techniques. Interesting to point towards future studies combining your concept with further or innovative techniques."

VERSION 2 – AUTHOR RESPONSE

Reviewer: 1
Dr. Chaitanya Vijaykumar Mahamuni, Agnel Charities' Father Conceicao Rodrigues Institute of Technology
Comments to the Author:

1. *The said corrections are made and the paper can be accepted for publication.*

Author's response: Thank you very much for your review and comments.

Reviewer: 2 Dr. Sam Sedaghat, University Hospital Heidelberg

Comments to the Author: Thank you for your changes that improved the manuscript significantly. However, the requested references have not been added to your manuscript. Also, I cannot find the requested perspective section:

7. *Please add a short future perspective and please discuss in a few words that future studies could deploy this strategy on more advanced imaging techniques for fracture detection like CT.*

Author's response: Thank you for this suggestion, however we would prefer to not to include a discussion/perspective section in this protocol paper, as they are not typically included at this stage until the findings of the study have become clear - we have already successfully published protocol papers for 2 related studies in BMJ Open without including a section of this type. We do appreciate the value of such sections however, and will plan to include one in the final paper resulting from the study.

8. *Please consider adding these recently published article to your references: https://doi.org/10.1016/j.ejrad.2024.111364 and https://doi.org/10.3390/diagnostics13172745. The one is on AI in spinal fractures, the other is on detecting fractures using novel imaging techniques. Interesting to point towards future studies combining your concept with further or innovative techniques."*

Author's response: Thank you for this suggestion - whilst we appreciate the signposting to these interesting papers co-authored by the reviewer, we note that they relate primarily to CT images rather than X-Rays so are not sure that they are strongly relevant to the study in hand. We will however certainly consider them in one of our other planned studies involving photon counting CT, and so are grateful to have been made aware of them.

Thank you very much again. We look forward to your feedback and hope you find the revised version suitable for publication in BMJ Open.
